# Effect of CO_2_ Flow Rate on the Extraction of Astaxanthin and Fatty Acids from *Haematococcus pluvialis* Using Supercritical Fluid Technology

**DOI:** 10.3390/molecules25246044

**Published:** 2020-12-21

**Authors:** Carolina Espinosa Álvarez, Renata Vardanega, Francisca Salinas-Fuentes, Jenifer Palma Ramírez, Waldo Bugueño Muñoz, Diana Jiménez-Rondón, M. Angela A. Meireles, Pedro Cerezal Mezquita, Mari Carmen Ruiz-Domínguez

**Affiliations:** 1 Laboratorio de Microencapsulación de Compuestos Bioactivos (LAMICBA) del Departamento de Ciencias de los Alimentos y Nutrición, Facultad de Ciencias de la Salud (FACSA), Universidad de Antofagasta, Avda, Universidad de Antofagasta 02800, Campus Coloso, P.O. Box 1240000 Antofagasta, Chile; carolina.espinosa@uantof.cl (C.E.Á.); francisca.salinas@uantof.cl (F.S.-F.); jenifer.palma@uantof.cl (J.P.R.); waldo.bugueno@uantof.cl (W.B.M.); diana.jimenez@uantof.cl (D.J.-R.); maria.ruiz@uantof.cl (M.C.R.-D.); 2 LASEFI-Department of Food Engineering, School of Food Engineering, University of Campinas (UNICAMP), R. Monteiro Lobato 80, 13083-862 Campinas, São Paulo, Brazil; maameireles@lasefi.com; 3 Entourage Phytolab, R. Tabapuã 111, 04533-010 São Paulo, São Paulo, Brazil

**Keywords:** astaxanthin, supercritical fluid extraction, microalgae, fatty acids

## Abstract

*Haematococcus pluvialis* is the largest producer of natural astaxanthin in the world. Astaxanthin is a bioactive compound used in food, feed, nutraceutics, and cosmetics. In this study, astaxanthin extraction from *H. pluvialis* by supercritical fluid extraction was evaluated. The effects of temperature (40 and 50 °C), pressure (40 and 50 MPa), and CO_2_ flow rate (2 and 4 L/min) were investigated. The results showed that the highest astaxanthin recovery was obtained at 50 °C/50 MPa and the CO_2_ flow rates evaluated had no significant effect. It was possible to achieve astaxanthin recoveries of 95% after 175 min for a CO_2_ flow rate of 2 L/min, and 95 min for CO_2_ flow rate of 4 L/min. The ω-6/ω-3 ratios obtained were similar in all conditions, reaching 0.87, demonstrating that the extracts from *H. pluvialis* by SFE are rich in unsaturated fatty acids (UFA) which increases their positive effects when used as a functional ingredient in food.

## 1. Introduction

**Figure 1:**
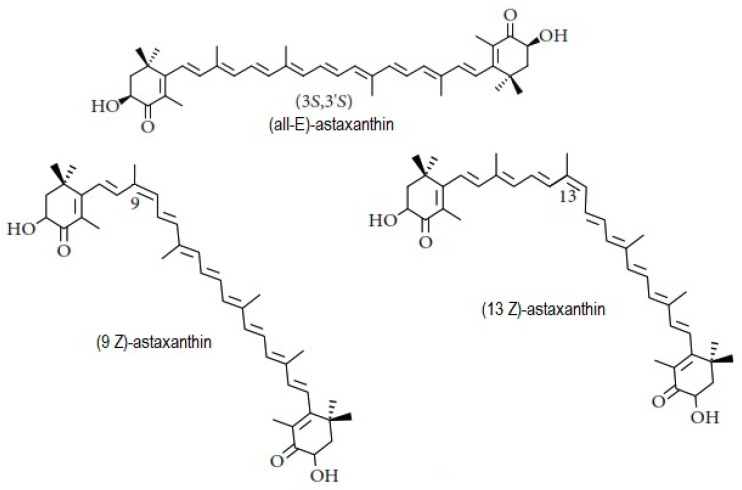
Structure of the geometrical isomers (all-E)-astaxanthin, (9Z)-astaxanthin, and (13Z)-astaxanthin.

The bioactive compound astaxanthin belongs to the carotenoid group, specifically of the xanthophyll sub-group [1,2]. A remarkable characteristic of this pigment is its red-orange color, and it is also considered as the most valuable compound obtained from *Haematococcus pluvialis* microalgae which is led it to present a wide use in the pharmaceutical, food, animal feed, and cosmetic industries [1,3,4,5]. Due to the structure of astaxanthin (3,3′-dihydroxy-*ß*,*ß*′-carotene-4,4′-dione; CAS no. 472-61-7; Mw = 596:8 g mol^−1^; ε_468 nm_ = 125 × 10^−3^ mol^−1^ in hexane), it has been described that it has an antioxidant capacity 65 times greater than vitamin C; it is 10 times more potent than β-carotene, canthaxanthin, zeaxanthin, and lutein; additionally, it is also 100 times more effective than α-tocopherol [3,4,5,6]. The *H. pluvialis* microalgae synthesizes mainly the 3S, 3′S isomer [7]. Astaxanthin also exists as geometrical isomers (E and Z) (Figure 1), depending on the configuration of the double bonds in the polyene chain. All-E-astaxanthin is the dominant isomer, although at least two isomers (9Z and 13Z) also occur in nature, depending on the host species and body part, and are also found in synthetic preparations [8].

Global market demands for naturally derived astaxanthin from algae were valued at more than $385 million in 2018 according to research reports recently published by Global Market Insights [9]. Synthetic astaxanthin has a 20-fold lower antioxidant capacity than the natural form and corresponds to 95% of the astaxanthin available in the market. Only natural astaxanthin is approved for human consumption by the Food and Drug Administration (FDA) [6,10,11]. *H. pluvialis* is the largest producer of natural astaxanthin in the world; however, it has been described that the astaxanthin accumulation in the microalgae promotes physical changes, going from a cell in mobile phase with green color to an immobile cell of red color (aplanospore), which is known to present a hard cell wall, that is also very resistant to physical and chemical alterations [12,13].

Carotenoid compounds are conventionally recovered by extraction techniques using organic solvents. Nevertheless, in recent years, severe restrictions have been proposed for the use of these solvents in industrial processes in favor of health, in addition to the impact of them on the environment [14]. Nowadays, there are conventional extraction methodologies for bioactive compounds that use organic solvents and that are being replaced by means of CO_2_ extractions, in subcritical and/or supercritical state [15]. Unlike organic solvents which leave potentially toxic residues in the extracts, supercritical CO_2_ is characterized by its high selectivity, by avoiding thermal damage to labile bioactive compounds, its non-toxicity and its properties with gentle critical temperature and pressure, 31.1 °C and 74.0 bar, respectively [16,17]. Therefore, it is considered a new alternative for the benign extraction of natural compounds, but the selection of operating conditions for specific applications continues to be an area of active research, since this will depend on the compound to be extracted [16,18].

Accordingly, the quality of the extracts obtained by supercritical CO_2_ is much higher than those provided by other extraction techniques, such as: Liquid-liquid extraction with organic solvents or steam distillation, since these methods can induce thermal degradation or leave residues of toxic organic solvent in the products [19]. Therefore, clean extraction techniques such as supercritical fluid extraction have been developed as an alternative to conventional methods and to recover BAC from plant materials or their wastes [20,21].

Several studies have also been reported on the supercritical fluid extraction (SFE) of astaxanthin from *H. pluvialis* [22,23,24] performed with and without the addition of ethanol as a co-solvent [23,25]. It has been established that the total amount of astaxanthin in the extract and its concentration are influenced by the pressure and temperature from supercritical extraction [26]. The aforementioned was demonstrated by Nobre et al. [27] who have studied the effect of different temperatures (40–60 °C) and pressures (200 and 300 bar), and by Machmudah et al. [23] who evaluated the effect of pressure (200 to 550 bar) and temperature (40 and 80 °C). Di Sanzo et al. [12] carried out a study where the effect of temperature (50 and 80 °C), pressure (100–550 bar), extraction time (20–120 min), and CO_2_ flow (3.62 and 14.48 g/min) were evaluated.

The combination of mechanical cell disruption and co-solvent extraction has been tested to speed up extraction and improve recovery, using ethanol [27,28], and vegetable oil [29] as co-solvent. However, the associated high capital and operating costs present barriers to large-scale production [30]. Discrepancies between literature reports present additional uncertainty. Process control and resolution at the cellular scale are essential to quantify the effectiveness of extraction processes and to inform the extraction of astaxanthin from microalgae based on supercritical CO_2_ on an industrial scale [31].

The aim of the present study was to investigate the effect of the variables temperature (40 and 50 °C) and pressure (40 and 50 MPa) on the total extraction yield, astaxanthin recovery, and fatty acids from of *H. pluvialis* dry biomass obtained by SFE.

## 2. Results and Discussion

### 2.1. Microalgae Material and Characterization

The proximate composition of whole *H. pluvialis* powder was expressed as % dry basis (d.b.), of biomass is presented in Table 1.

As can be seen, the mean values of the proximal composition of *H. pluvialis* powder used varies according to the origin of the biomass used, especially regarding the lipids content. It can be associated with the growing conditions and stress factors that strongly affects the availability of nutrients, and for instance, the composition of the microalgae [6,34,35].

The initial moisture content of powdered microalgae biomass is an important parameter to be considered in SFE processes because the lack of water in the biomass causes a contraction of the cell structure, complicates the diffusion and penetration of the extraction solvent, resulting the total extraction yield decreases. On the contrary, the water excess can create an additional barrier for the transport and generate the co-extraction of polar substances, reducing the selectivity [36]. Reyes et al. [37] reported moisture content of 3.3 ± 0.2% (d.b.) for SFE of astaxanthin from *H. pluvialis*, similarly to that used in the present study.

The particle size is another important parameter in SFE since the porosity and physical properties of the extraction bed are affected directly by this condition, hence the particle diameter is inversely proportional to the total extraction yield [38,39]. In the present study the mean particle size of the *H. pluvialis* powder was Dp = 0.32 ± 0.01 mm. Priyanka [40] reports that if the particle size is too small, channeling of fluids can occur within the fixed bed resulting in inhomogeneous extraction. On the other hand, for larger particles, the cell walls do not break where the oil is trapped. Therefore, a moderate particle size of around 0.45 mm is appropriate to maximize oil yield [41].

### 2.2. Total Yield and Extracts Composition

**Figure 2:**
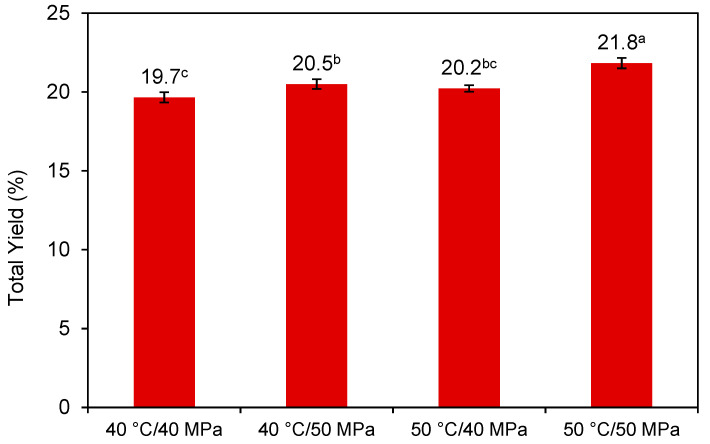
Total extraction yield obtained from *H. pluvialis* SFE at different pressure and temperature conditions. Different superscript letters ^a–c^ indicate statistically significant differences (*p* < 0.05).

The total yields of the extracts obtained from *H. pluvialis* obtained by SFE under different conditions of temperature and pressure are presented in Figure 2. The total yields were significantly affected by the combinations of temperature and pressure being the highest total yield (21.8 ± 0.3%, d.b.) obtained at the highest temperature and pressure (50 °C/50 MPa) while the lowest total yield corresponded to the condition of 40 °C/40 MPa, being 19.7 ± 0.3% (d.b). Increasing pressure at constant temperature improved the total yield, which is related to the CO_2_ density increase that ranged from 0.956 to 0.991 g/mL for pressures of 40–50 MPa at 40 °C, and from 0.923 to 0.962 g/mL for the pressures of 40–50 MPa at 50 °C.

The content, yield and astaxanthin recovery of the extracts obtained by SFE are shown in Table 2. As can be seen, the different temperature and pressure conditions had a significant effect on the content and yield of astaxanthin according to the Tukey’s Test performed between the mean values. The best extraction condition, statistically significant (*p* < 0.05) to the rest of the processes, was 50 °C/50 MPa, since it presented the highest astaxanthin concentration in the extract (84 ± 6 mg of astaxanthin/g extract) as well as the of the highest astaxanthin yield (19 ± 1 mg of astaxanthin/g of biomass) and corresponded to a total astaxanthin recovery of 80.0%, since the *H. pluvialis* biomass used in the present study had 23.3 ± 0.1 mg of astaxanthin/g of biomass.

The processes whose conditions were 40 °C/50 MPa and 50 °C/40 MPa did not present statistical differences (*p* < 0.05) between them (Table 2); nevertheless, the process whose condition was 50 °C/40 MPa did not have significant differences (*p* < 0.05) with respect to that of 40 °C/40 MPa according to Tukey’s Test. It can be stated that these two processes had the lowest content, yield and astaxanthin recovery, which is observed in the values shown in Table 2.

The temperature and pressure increases favored the both astaxanthin content and astaxanthin yield, despite the changes in the CO_2_ density. It is well known that the pressure increase at constant temperature increases the CO_2_ density, thus favoring the solvation powder of the CO_2_. In this case, was also observed the positive effect of the temperature on the solubilization of astaxanthin in the CO_2_, despite the CO_2_ density reduction with the temperature increase at constant pressure. It suggests that the effect of the vapor pressure with the temperature had a more significant effect than the reduction of the CO_2_ density. However, it is worth mentioning that temperature increases over 50 °C can reduce the astaxanthin recovery due to thermal degradation of subunits of astaxanthin as extensively reported in the literature [33,42,43].

**Figure 3:**
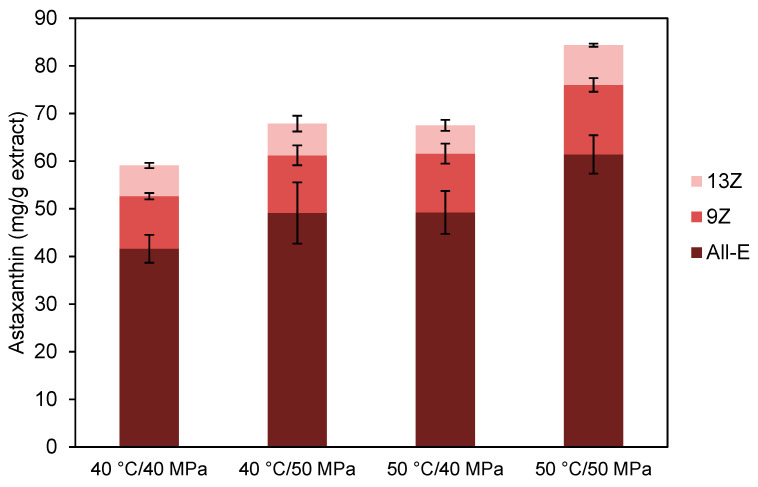
Astaxanthin isomers distribution (All-E, 9Z, and 13Z) in the extracts obtained from *H. pluvialis* by SFE under different extraction conditions.

Di-Sanzo et al. [12] reported that the highest astaxanthin recovery (98.6%) was observed at 50 °C and 55 MPa while at 65 °C the astaxanthin recoveries fall up to 36%. The total astaxanthin content reported in Table 2 was the sum of the 3 isomers quantified in the extracts (E-astaxanthin, 9Z-astaxanthin and 13Z-astaxanthin), which distribution is the extracts is presented in Figure 3. It can be seen that the proportion between the isomers was not affected by the extraction conditions evaluated been 72 ± 1% E-astaxanthin, 18.0 ± 0.6% 9Z-astaxanthin, and 9.9 ± 0.9% 13Z-astaxanthin. The geometric isomers of astaxanthin in *H. pluvialis* generally occur in a range of: All-E (88–90%), 9Z (7–8%), and 13Z (3–5%) [44]. However, the all-E isomer and the Z isomers can exchange with each other under certain conditions, such as high temperature, exposure to light, and the presence of acids and organic solvents [45].

### 2.3. Fatty Acids Composition Analysis

The fatty acid profile of the extracts from supercritical *H. pluvialis* is shown in Table 3. The main fatty acids found in the extractions were α-linolenic (C18:3n3, ALA), linoleic acid (C18:2n6c, LA), oleic (C18:1n9c) and palmitic (C16:0) acids. They were almost 95% of total of fatty acids (equivalent to an average of 484.5 mg/g extract) present in *H. pluvialis* biomass. These fatty acids did not have statistically significant differences (*p* < 0.05) between them under the different conditions of SFE extractions according to the Tukey’s Test.

Additionally, the fatty acids profile of *H. pluvialis* extracts also showed eicosapentaenoic acid (C20:5n3, EPA) with values ranging from 4 ± 2 to 5.2 ± 0.7 mg/g extract (equivalent to 0.82–1.03% total fatty acids) and arachidonic acids (C20:4n6, ARA) from 5 ± 2 to 9 ± 3 mg/g extracts (equivalent to 1.02–1.59% total fatty acids). Both ω-3 and ω-6 fatty acids are described for their benefits properties in the human health. Particularly, EPA is well-known for its positive effects on fetal development, cardiovascular function, and the prevention of Alzheimer’s disease contributing to the mental health of adults [46]. On the other hand, ARA plays a crucial role in the regulation of blood pressure and in the pathobiology of hypertension and diabetes mellitus along with the presence of potent anti-inflammatory metabolites [47]. Ruiz-Domínguez et al. [48] also found EPA and ARA in similar proportions to our results (1.28% and 0.43% of total fatty acids, respectively) for extracts from *H. pluvialis* obtained by SFE. The results presented by Ruiz-Domínguez et al. [48] demonstrated a high concentration of unsaturated fatty acids (UFA) which represents the sum of monosaturated and polyunsaturated fatty acids (MUFA+PUFA) that was approximately 78% of total fatty acid. The present study also showed relevant content of UFA in the range from 78 to 79% of total fatty acids without significant differences (equivalent to 381–448 mg/g extract).

The total fatty acids content in the extracts ranged from 482 ± 72 to 568 ± 78 mg/g extract without significant statisticaly differences (*p* < 0.05) between the different extraction conditions. These data resulted in a fatty acid yield around 10.0% of *H. pluvialis* biomass and the highest fatty acids yield (11.4 ± 0.7%) was obtained at 40 °C/50 MPa. Therefore, there were only significant statistical differences (*p* < 0.05) in C18: 3n6-ɣ-Linolenic acid (GLA) between the process conditions of 40 °C/40 MPa and 40 °C/50 MPa, but this was not the case between 40 °C/40 MPa and 50 °C/50 MPa and neither between 50 °C/40 MPa and 50 °C/50 MPa.

Di-Sanzo et al. [12] also studied the extraction of fatty acids from *H. pluvialis* in red phase using SFE. The maximum recovery of fatty acids was attained was 93.25% (equivalent to 21.41 mg/g biomass) at 65 °C and 55 MPa (550 bars), with a CO_2_ flow rate of 3.62 g/min. Here, we presented a fatty acid yield about five-fold superior to those results. Moreover, it was observed that lower temperature (40 °C) favored the fatty acids content at high pressure (50 MPa) similarly to the pressure of 55 MPa used by Di-Sanzo et al. [12]. Among fatty acid profiles, their results were consistent with these works (equal to ~60% of PUFA of total fatty acids) because PUFAs were extracted with the highest recovery.

Finally, the ratio of ω-6/ω-3 was calculated and is presented in Table 3. This ratio is crucial in maintaining the overall health of the human population [49]. In general, a lower ratio of ω-6/ω-3 fatty acids is more desirable for reducing the risk of many chronic diseases [50] of high prevalence in Western societies, as well as in the developing countries who are rapidly adopting Western dietary habits [51,52]. The ω-6/ω-3 ratio found in the present study was around 0.87, suggesting that the extracts from *H. pluvialis* rich in UFA could increase its positive effects when used as a functional ingredient in foods.

These results are in agreement with the literature that reports that the solubility of lipids in supercritical CO_2_ depends on their chemical composition, besides pressure and temperature. When C16 fatty acids prevail in triglycerides, the solubility of extracted oil is higher than the solubility of vegetable oils with prevailing C18 fatty acids, for which a correlation with extraction temperature and solvent density was published by del Valle et al. [53]. The main constituents of the *H. pluvialis* fatty acid profile are esterified to C18 and C16 fatty acids in lipid vesicles [54,55]. As the C18 fatty acids prevail, the extract solubility in supercritical CO_2_ can be expected to be similar to that of common vegetable oils, where also C18 fatty acids prevail. In this way, the solubility of the oil increases with increasing pressure due to the increase of the solvation power of CO_2_, allowing a higher permeability of the solvent into the matrix [56].

### 2.4. Kinetic Study

**Figure 4:**
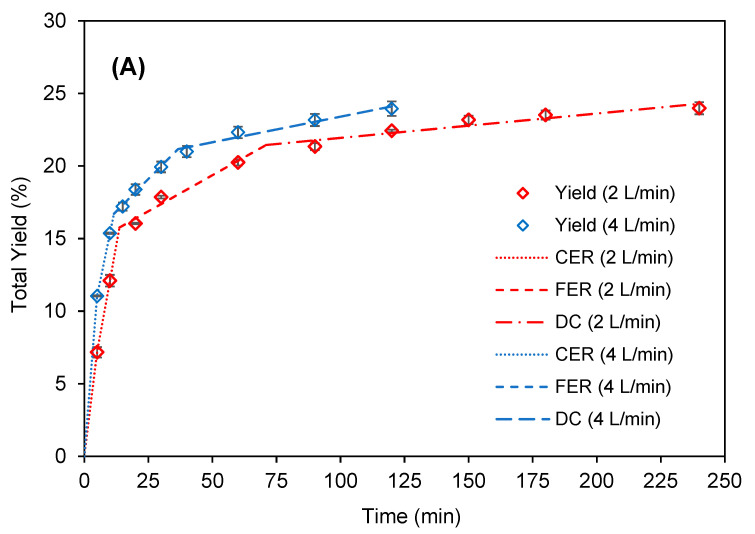

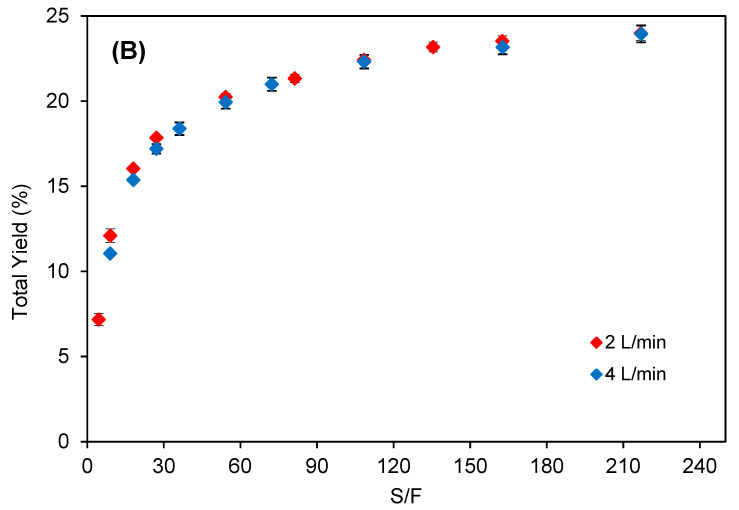
Overall extraction curve of *H. pluvialis* obtained by SFE at 50 °C 50 MPa with different CO_2_ flow rates (2 and 4 L/min). (**A**) Data plotted in function of extraction time and (**B**) in function of S/F ratio.

**Figure 5:**
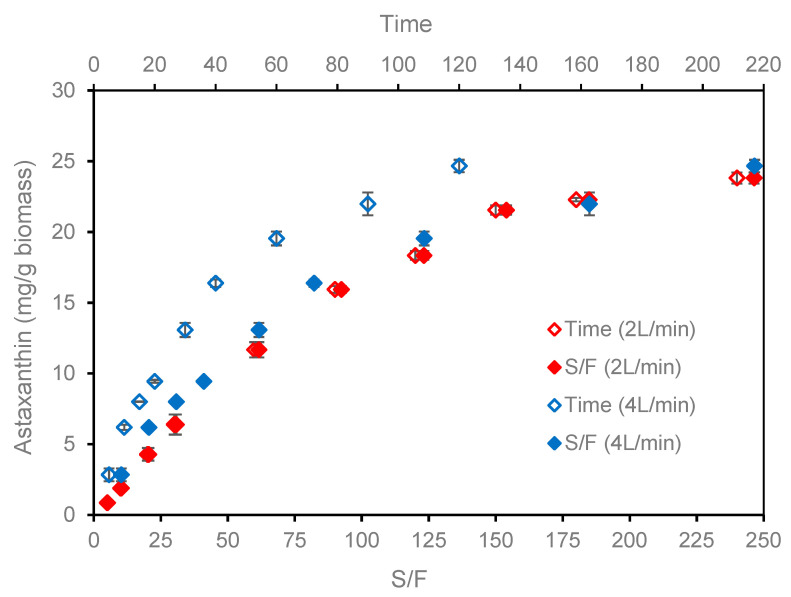
Astaxanthin yield from *H. pluvialis* extracts obtained by supercritical extraction at 50 °C/50 MPa, with different flow rates (2 L/min and 4 L/min).

The overall extraction curves (OECs) were obtained at 50 °C/50 MPa, because this condition favored the maximum astaxanthin recovery. The OECs were constructed using CO_2_ flow rates of 2 and 4 L/min (3.62 and 7.24 g/min, respectively) keeping the S/F ratio (217) to evaluate its effect on the kinetic behavior. Figure 4 presents the OECs for the total extraction yield while Figure 5 presents the results regarding the astaxanthin yield.

An accumulated yield of 24.3 ± 0.5% (d.b.) and 24.1 ± 0.4% (d.b.) was obtained for CO_2_ flow rates of 2 L/min and 4 L/min, respectively, demonstrating that the CO_2_ flow rate did not affect the total extraction yield of *H. pluvialis* at the same S/F ratio although affected the kinetic parameters (Table 4).

The OECs followed the SFE kinetic behavior described by Meireles [57] and Jesus et al. [58]. The extraction process began with the constant extraction rate (CER) period, characterized by the removal of easily extractable compounds by the solvent, which was mainly controlled by the convective mass transfer in the fluid film around the powder particles. After the CER period, the transition period began with a reduced extraction rate, in which the extraction rate was controlled by mass transfer mechanisms through both convection and diffusion. This period is commonly called the falling extraction rate (FER) period. When the easily accessible solute became scarce in the microalgae matrix, intraparticle diffusion became the main mass transfer mechanism during SFE, and thus the OEC assumed a typical shape diffusion curve with reduced extraction rate, called as the diffusion controlled (DC) period.

The different flow rates did not affect the behavior of the *H. pluvialis* biomass extraction curves, for the evaluated conditions. This indicates that the extraction time and the CO_2_ flow can be adapted according to the limit conditions of the equipment, without causing damage to the process performance. It is noteworthy that the performance curves are not affected by flow rate so the extraction process is limited by solubility and not by diffusion (Figure 4).

The calculated t_CER_ were 13.71 and 11.56 min with extract yield of 15.8% and 16.7% corresponding to total extract recoveries of 55.7% and 66.6% for CO_2_ flow rates of 2 L/min and 4 L/min, respectively. This is in agreement with a previous study that reported the recovery between 50% and 90% in the CER period [59]. In relation to astaxanthin (Figure 5) the CER period corresponds to 2.8 and 6.8 mg astaxanthin/g biomass (astaxanthin recovery of 11.9 and 29.0%) for CO_2_ flow rate of 2 L/min and 4 L/min, respectively. Although the t_CER_ was similar for the both CO_2_ flow rates, the astaxanthin yield was 2.4-fold higher when the CO_2_ flow rate was doubled to 4 L/min.

The calculated t_FER_ was 71.0 and 36.6 min for the extraction yields of 21.4% and 21.2%, for CO_2_ flow rates of 2 L/min and 4 L/min, respectively. In the FER period, both CO_2_ flow rates resulted a total extract recovery 86.1%. Figure 5 present the extraction yield in function of the S/F ratio, where it can be seen that the OECs for both CO_2_ flow rates are practically overlapped, and the S/F of the FER period was 64.1 and 66.1 for the CO_2_ flow rates 2 L/min and 4 L/min, respectively. This suggests that the solubility of the extracts from *H. pluvialis* is not affect by the CO_2_ flow rate. It explains why the t_FER_ for the CO_2_ flow rate of 2 L/min was almost the double of that for 4 L/min.

Di-Sanzo et al. [12] also evaluated the effect of the CO_2_ flow rate (2 L/min and 8 L/min) on the astaxanthin recovery from *H. pluvialis* and observed that the astaxanthin recovery was affected by the CO_2_ flow rate. According to them, the lower the CO_2_ flow rate, the greater astaxanthin recovery. An astaxanthin recovery of approximately 90% was achieved in the first and second extraction cycles (40 min), with a CO_2_ flow rate of 2 L/min and approximately 35% with a flow rate four times higher (8 L/min). However, it is worth to mention that the highest CO_2_ flow rate evaluated by these authors was 8 L/min while in the present study the highest value was 4 L/min, which demonstrates that it is possible to increase the CO_2_ flowrate in order to enhance the process productivity, but there is an optimal region that this increase does not affect the solubility of the compounds in the solvent.

The M_CER_ and M_FER_ values represent the extraction rate of the CER and FER periods respectively [58], with values of 0.039 and 5.0 × 10^−3^ g extract/min and 0.056 and 7.4 × 10^−3^ g extract/min for CO_2_ flow rates of 2 and 4 L/min. This represents 10.45 and 8.50 mg astaxanthin/g biomass with total recovery of astaxanthin of 56.7 and 65.4% (Figure 5), being 1.15 times higher for a CO_2_ flow rate of 7.24 g/min obtained in only 36.57 min.

To obtain total recovery of astaxanthin ≈ 85%, which are adequate values for industrial processes, the times necessary for CO_2_ flows of 2 and 4 L/min would be 134 and 64 min, representing a S/F ratio of 121 and 116, respectively, which is equivalent to savings of 4.5% for the higher CO_2_ flow rate and other associated savings in direct and indirect costs. If greater efficiency is required in the industrial process; that is, total recovery of astaxanthin ≈ 95%, the times required for flow rates of 2 L/min and 4 L/min would increase to 175 and 95 min, respectively. In this case, the S/F would be 158 for 2 L/min and 171 for 4 L/min, representing CO_2_ savings of 8.6% in favor of the lower CO_2_ flow rate. In the same way, these expenditure returns would be conditioned with the production process, which would involve the CO_2_ costs and its recovery, electricity, and other direct and indirect costs, for which a specific study would have to be performed. As is known, to the extent that the process is scaled up, there must be a compromise between costs and benefits of the production system.

Molino et al. [33] conducted research on the astaxanthin extraction from *H. pluvialis* by SFE, in which ethanol was used as a co-solvent. CO_2_ and ethanol flow rates were kept constant at 2 L/min and 1 mL/min, respectively. The results showed that the highest astaxanthin recoveries were found at 50 °C/55 MPa, and extraction time of 80 min with a recovery of 94.5% of astaxanthin.

## 3. Material and Methods

### 3.1. Microalgae Material and Characterization

*H. pluvialis* dry biomass disrupted (Astaxanthin: Food grade powder), was kindly provided by Atacama BioNatural Products S.A. (Iquique, Chile). To avoid degradation, this substrate was placed under reduced pressure in a tightly sealed aluminum bags and stored at −20 °C until use.

All chemicals used in this study were of analytical grade, except for those used for HPLC and GC analyses which were of chromatographic grade, methanol, acetonitrile, ethyl acetate, dimethyl sulfoxide, water, and n-hexane, were acquired from Merck (Darmstadt, Germany). Astaxanthin standard (purity > 95%) was obtained from Dr. Ehrenstorfer GmbH (Augsburg, Germany). Cholesterol esterase from *Pseudomonas fluorescens* C9281 (lyophilized powder 4 units/mg protein) was procured from Sigma-Aldrich (Santiago, Chile). For fatty acid identification and quantification, a standard fatty acid methyl ester (FAME) mix, C4–C24, by Supelco Analytical (St. Louis, MO, USA) was used, and tripentadecanoin > 99% (Nu-Check Pre, Inc., Elysian, MN, USA) was used as the internal standard. Commercial grade liquid carbon dioxide (99.9% purity) contained in a cylinder with an eductor tube, was acquired from Indura Group Air Products (Santiago, Chile).

The particle size distribution was determined using a Ro-Tap test shaker sieve (RX-29, WS Tyler, Mentor, OH, USA) with mesh sizes of 35, 70, 100, 140, 200, and 230. After 15 min shaking, the material retained in each sieve and the bottom pan were weighed and recorded. The mean particle diameter (Dp) of *H. pluvialis* powder was determined according to ASAE methodology [60] using Equation (1).

(1) $$D_{p}=\exp\left\{\frac{\sum_{i=1}^{n}[\sqrt{W_{i}\cdot \log\left(d_{i}\cdot d_{i+1}\right)}]}{\sum_{i=1}^{n}W_{i}}\right\}$$

where $D_{p}$ is the mean particle diameter (mm); $d_{i}$ is the diameter of the sieve opening i (mm); $d_{i+1}$ is the diameter of the sieve opening above sieve i (mm); $W_{i}$ is the retained mass (g); n is the total numbers of fractions.

The *H. pluvialis* microalgae powder was characterized to determine its proximal composition: the moisture content was determined by method AOAC No. 920.151 [61], the ash content was determined according to the method No. 923.03 of the AOAC [61], and the lipid content was quantified by method No. 945.16 of the AOAC [61]. Meanwhile, for the determination of protein content, method AOAC No. 970.22 [61] was used with a conversion factor of 6.25. Finally, carbohydrate content was calculated by difference. All analyses were performed in duplicate.

### 3.2. Supercritical Fluid Extraction (SFE)

**Figure 6:**
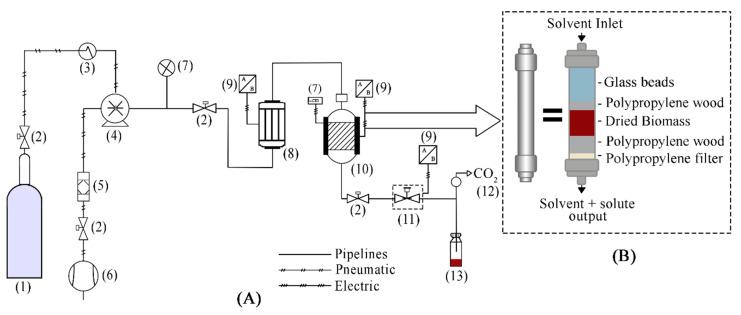
(**A**) Diagram of the SFE equipment (Applied Separations, Spe-ed, Allentown, PA, USA). (1) CO_2_ cylinder; (2) Blocking valve; (3) Cooling bath; (4) CO_2_ pump; (5) Air filter; (6) Air compressor; (7) Pressure gauge; (8) Heater; (9) Temperature controller; (10) Extraction vessel and oven: (11) Micrometering valve; (12) Flow meter; (13) Sample collection. (**B**) Schematic diagram of the extraction vessel showing the way in which each of the elements that comprise it are arranged.

The SFE conditions evaluated to recover the astaxanthin from *H. pluvialis* were temperatures of 40 and 50 °C and pressures of 40 and 50 MPa. The SFE runs were carried out in a commercial SFE unit (Spe-ed SFE Helix, Applied Separations, Allentown, PA, USA) schematically showed in Figure 6. Briefly, the Spe-ed SFE Helix unit contains a pneumatic pump for CO_2_ (CO_2_ pump) and an HPLC pump (modifier pump) that enables using co-solvents with the CO_2_, and a chiller to reduce the CO_2_ temperature. The system also presents a heat exchanger that enables the pre-heating of CO_2_ before entering the extraction vessel. The Spe-ed SFE Helix permits the use of extraction vessels with volumes from 24 mL to 1 L, which are heated by an electrical jacket (temperature controller). In the exit of the extraction vessel there is a blocking valve (exit valve) followed by a micrometering valve that controls the CO_2_ flow rate. The CO_2_ flow rate is measured in an electronic flowmeter installed in the exit of the sample collection flask.

The 24 mL extraction vessel with dimensions of 1.50 cm of diameter and 13.50 cm of height was packed with 2 g of astaxanthin powder, occupying a volume of 2.46 mL (10% of the total volume of the vessel) resulting a bed density of 0.813 g/mL. The empty space of the extraction vessel was filled with glass beads of 1.0 mm. The vessel was assembled into the heat jacket at a selected temperature and kept for 15 min until the desired temperature was stabilized.

The CO_2_ was pumped in the bed until reach the desired pressure and was kept for 5 min in a static period. After this time, the micrometric valve was opened at a constant CO_2_ flow rate to collect the sample. The CO_2_ flow rate was 2 L/min (3.62 g/min) for 60 min reaching a solvent to feed ratio (S/F) of 108.6. For the kinetic study, an overall extraction curve (OEC), was obtained at the extraction condition that resulted the highest total extraction yield (50 °C and 50 MPa). For this, 4.0 g of *H. pluvialis* were used and CO_2_ flow rates of 2 and 4 L/min (3.62 and 7.24 g CO_2_/min, respectively) up to S/F 108.6 for both kinetic curves.

### 3.3. Spline Model

The overall extraction curves (OEC) were fitted to a spline model (Equation (2)) using three straight lines, presented in Equations (3)–(5). Each fitted line represents an extraction stage related to a mass transfer mechanism: CER and FER periods, which represents the step for which both convection and diffusion in the solid substratum controls the process, and DC period, as described by Meireles [57]. In the CER period, the rate of mass transfer for the CER period (*M_CER_*) as well as the time corresponding to the interception of the two lines (*t_CER_*) was computed from the spline. Similar procedure was followed for the FER period, and finally the DC stag was indicated. The spline was fitted using the PROC REG and PROC NLIN through the SAS University Edition Software. Finally, the fitted data from Equation (6) were plotted using Microsoft Excel 2016. The mass ratio of solute in the supercritical phase at the equilibrium cell outlet (Y_CER_) was obtained by dividing M_CER_ by the mean solvent flow rate for the CER period.

(2) $$y=m_{\mathrm{Ext}}=\left(b_{0}-\overset{i=N}{\underset{i=1}{\sum }}C_{i}a_{i+1}\right)+\overset{i=N}{\underset{i=1}{\sum }}a_{i}t$$

(3) $$y=m_{\mathrm{Ext}}=b_{0}+a_{1}t\;for\;t\leq t_{\mathrm{CER}}$$

(4) $$y=m_{\mathrm{Ext}}=b_{0}-t_{\mathrm{CER}}a_{2}+\left(a_{1}+a_{2}\right)\;t\;for\;t_{\mathrm{CER}}<t\leq t_{\mathrm{FER}}$$

(5) $$y=m_{\mathrm{Ext}}=b_{0}-t_{\mathrm{CER}}a_{2}-t_{\mathrm{FER}}a_{3}+\left(a_{1}+a_{2}+a_{3}\right)\;t\;for\;t_{\mathrm{FER}}<t$$

where y = response variable = $m_{Ext}$ is the mass of extract; $a_{i}$ (i = 0, 1, 2, 3); linear coefficients of lines; t = time (min); $t_{\mathrm{CER}}$ = CER time (min); $t_{\mathrm{FER}}$ = FER time (min). $C_{I}$ for i = 1, 2 are the intercepts of these lines (for instance: $C_{1}$ is the intercept of the first and second lines, and $C_{2}$ is the intercept of the second and three lines.

Using the adjusted parameters, the Y_CER_ and Y_FER_ achieved in t_CER_ and t_FER_ were calculated. Then, recoveries were calculated at each time, according to Equation (6):

(6) $$Recovery\;\left(\%\right)=\frac{y_{t}}{y_{final\;time\;OEC}}\;(100)$$

### 3.4. Astaxanthin Quantification by High Performance Liquid Chromatography (HPLC)

The samples were purified to astaxanthin bioactive compound by the enzymatic hydrolysis method. Firstly, the preparation of samples was carried out according to Cerezal et al. [62] with some modifications. For that, 10 mg of sample were mixed with 2 mL of dimethyl sulfoxide, saturated brine solution n-hexane and 3 mL of acetone. This mixture was stirred for 15 s and separated in two layers by centrifugation (3500 rpm, 3 min). The upper layer (carotenoids and n-hexane) was transferred to a 10 mL centrifuge tube which contained 1 g of Na_2_SO_4_. These steps were repeated until the upper layer was colorless. Then, the upper solvent and carotenoid layer were removed from the tube and transferred to a 50 mL flask and the solvent was evaporated at 45 °C to dryness with a rotary evaporator (Jiangsu Zhengji Instruments Co., Ltd. Jiangsu, China). Immediately, the carotenoids were dissolved in acetone until its absorbance at 471 nm ranging from 0.8 to 1.2 in a spectrophotometer (Shimadzu, Kyoto, Japan).

Secondly the enzymatic hydrolysis was carried out. For that, 3 mL of carotenoid solution were mixed with 3 mL of Esterase Cholesterol in solution with Tris-HCl and then was stirred at 300 rpm, 45 min, 37 °C in a thermo shaker for microtube (MRC, Holon, Israel). Subsequently, 2 mL of n-hexane were added and the solution was mixed for 30 s and separated into two layers by centrifugation (3000 rpm, 3 min). The upper layer was placed in a test tube with 1 g of Na_2_SO_4_ to eliminate any residue of water. Next the upper layer was transferred to another tube and N_2_ was injected to dry the sample. Then, 3 mL of acetone were added and absorbance was measured at 471 nm.

Finally, to quantify the astaxanthin content, a HPLC system, model 7100 (Merck Hitachi LaChrom, Tokyo, Japan) equipped with three pumps (flow rate of 1 mL/min) and a UV-Vis detector was used at 471 nm. The samples (20 µL) were injected into a capillary column (C18, 250 × 4.6 mm, 5 µm, Restek, Bellefonte, PA, USA). The mobile phase consisted of solvent A (ethyl acetate), and solvent B (acetonitrile water 90:10). The gradient procedure was (100% B for 10 min; 50% B for 4 min; 40% B for 2 min and 100% B for 1 min. The identification of astaxanthin was achieved by comparison of the retention times with reference standards. Fresh standard solutions (1 to 20 ppm of astaxanthin) were prepared and injected to produce the standard astaxanthin curve. The calculation of the free astaxanthin concentration in the samples was performed using the standard curve which was found to be linear over the required range (1–25 µg/mL; R^2^ = 0.9971). Furthermore, astaxanthin esters were also calculated with few modifications [63,64]. All analyses were performed in triplicate.

### 3.5. Fatty Acids Composition Analysis by Gas Chromatography (GC)

The samples were converted to methyl esters (FAME) by trans-esterification method according to ISO 5509:2000. Briefly, 20 mg of astaxanthin powder and extracts were mixed with 300 μL of KOH 2M in methanol and 3 mL of hexane. This mixture was stirred continuously for 2 min and then left in the dark for 1 h. After this period, the supernatant was recovery and filtered for injection.

In order to analyze the fatty acid composition, a gas chromatograph (GC-FID, Shimadzu, GC 2010, Kyoto, Japan) equipped with flame ionization detector (FID) and a split/splitless injector was used. The samples (1 µL) were injected into a capillary column (Rtx-2330, 30 m × 0.32 mm i.d. × 0.20 μm film thickness, Restek, State College, PA, USA). The injector temperature was maintained at 250 °C in the split mode with split ratio of 1:20 and nitrogen was used as the carrier gas at a constant flow rate of 0.962 mL/min. The chromatographic separation was carried out according to (Medina-Pérez et al. [65] where the oven temperature program stated at 80 °C for 5 min, increased by 4 °C/min to 165 °C for 2 min, increased at 2 °C/min to 180 °C for 5 min, increased by 2 °C/min to 200 °C for 2 min, increased by 4 °C/min to 230 °C for 2 min, and finally increased by 2 °C to 250 °C for 2 min, whit a total run time of 72 min. The detector temperature was 280 °C. Individual FAMEs were identified by comparing their retention times with those of mixed FAME standards (FAME Mix C_4_–C_24,_ Supelco Analytical, St. Louis, MO, USA) and quantified by comparing their peak area with those of mixed FAME standards.

### 3.6. Statistical Analysis of Samples

An analysis of variance (ANOVA) was performed using the software Minitab16^®^ (Minitab Inc., State College, PA, USA) to evaluate the effect of the operational conditions on the total extraction yield, astaxanthin yield, astaxanthin recovery and fatty acids composition of the extracts of *H. pluvialis.* The significant differences at the confidence level of 95% (*p*-value ≤ 0.05) were evaluated by Tukey’s procedure.

## 4. Conclusions

This work allowed to find the best operating parameters for the recovery of astaxanthin and fatty acids from *H. pluvialis* by SFE. The results demonstrated that, at 50 °C/50 MPa, it is possible to achieve astaxanthin recoveries of 95% after 175 min for a CO_2_ flow rate of 2 L/min, and 95 min for CO_2_ flow rate of 4 L/min. Furthermore, the results suggested that in range of CO_2_ flow rates evaluated, this parameter had no significant effect on the astaxanthin recovery. Although the lowest extraction time is preferred for industrial applications, higher precision work will be required when carrying out a scale up at a pilot or industrial level, where it will be necessary to take into account the direct and indirect costs of the set of unit operations of the process.

## Figures and Tables

**Table 1:** Proximal composition of *H. pluvialis* used in the present study compared with the literature.

Component	Content (%, d.b.)		
	This Study	Other Studies	
		[32]	[33]
Protein	30.9 ± 0.4	27.0	25.7
Lipids	28.6 ± 0.3	39.7	2.6
Ash	13.0 ± 2.0	2.8	4.0
Moisture	3.8 ± 0.4	7.1	7.0
Carbohydrates	27.3	28.1	54.5

**Table 2:** Content, yield and astaxanthin recovery of extracts from *H. pluvialis* biomass obtained by SFE under different extraction conditions.

SFE Condition	CO_2_ Density (g/mL)	Content (mg Astaxanthin/g Extract)	Yield (mg Astaxanthin/g Biomass)	Recovery (%)
40 °C/40 MPa	0.956	59 ± 3 ^c^	11.6 ± 0.4 ^c^	49.7
40 °C/50 MPa	0.991	68 ± 5 ^b^	13.9 ± 0.8 ^b^	59.6
50 °C/40 MPa	0.923	68 ± 7 ^b c^	13.2 ± 0.9 ^b^	56.4
50 °C/50 MPa	0.962	84 ± 6 ^a^	19 ± 1 ^a^	80.0

Means followed by the same superscript letter (a,b,c) within a column are not significantly different at *p* < 0.05 level (Tukey’s test).

**Table 3:** Main fatty acids (mg/g extract) presents in the *H. pluvialis* extracts by SFE under different conditions.

Fatty Acids	T (°C)	40		50	
	P (MPa)	40	50	40	50
C14:0-Myristic acid		2.83 ± 0.02 ^a^	2.9 ± 0.8 ^a^	3 ± 1 ^a^	2 ± 1 ^a^
C16:0-Palmitic acid		95 ± 4 ^a^	92 ± 13 ^a^	110 ± 15 ^a^	96 ± 13 ^a^
C16:1-Palmitoleic acid		3 ± 1 ^a^	2.8 ± 0.2 ^a^	3.5 ± 0.1 ^a^	2.9 ± 0.6 ^a^
C18:0-Stearic acid		7.3 ± 0.7 ^a^	6.7 ± 0.3 ^a^	7 ± 2 ^a^	7.6 ± 0.5 ^a^
C18:1n9c-Oleic acid		98 ± 2 ^a^	93 ± 17 ^a^	108 ± 15 ^a^	91 ± 15 ^a^
C18:2n6c-Linoleic acid (LA)		132 ± 1 ^a^	126 ± 17 ^a^	145 ± 20 ^a^	131 ± 15 ^a^
C18:3n6-ɣ-Linolenic acid (GLA)		2.7 ± 0.3 ^b^	2 ± 1 ^b c^	6 ± 1 ^a^	1.2 ± 0.6 ^c^
C18:3n3–α-Linolenic acid (ALA)		155 ± 4 ^a^	147 ± 24 ^a^	170 ± 21 ^a^	149 ± 23 ^a^
C20:4n6-Arachidonic acid (ARA)		6 ± 2 ^a^	5.0 ± 0.2 ^a^	9 ± 3 ^a^	5 ± 2 ^a^
C20:5n3-Eicosapentaenoic acid (EPA)		5.2 ± 0.7 ^a^	4.4 ± 0.3 ^a^	5 ± 2 ^a^	4 ± 2 ^a^
SFA—Saturated Fatty acids		105 ± 4 ^a^	101 ± 13 ^a^	121 ± 18 ^a^	106 ± 15 ^a^
MUFA-Monosaturated Fatty acids		101.8 ± 0.7 ^a^	96 ± 17 ^a^	112 ± 15 ^a^	94 ± 16 ^a^
PUFA-Polyunsaturated Fatty acids		300 ± 3 ^a^	285 ± 42 ^a^	336 ± 45 ^a^	290 ± 43 ^a^
Total FA (mg/g extract)		507 ± 9 ^a^	482 ± 72 ^a^	568 ± 78 ^a^	490 ± 73 ^a^
Total FA Yield (% g/g biomass)		10.0 ± 0.9 ^a^	9.9 ± 0.7 ^a^	11.4 ± 0.7 ^a^	10.7 ± 0.6 ^a^
Omega 6/Omega 3		0.86	0.87	0.88	0.89

Means followed by the same superscript letter (a,b,c) within a row are not significantly different at *p* < 0.05 level (Tukey’s test). Abbreviations: T: Temperature (°C), P: Pressure (MPa); FA: fatty acids.

**Table 4:** Adjusted parameters of spline model to SFE from *H. pluvialis* biomass at 50 °C/50 MPa and CO_2_ flow rates of 2 and 4 L/min.

Parameters	Stages of the Overall Extraction Curve					
	CO_2_ Flow Rate (2 L/min)			CO_2_ Flow Rate (4 L/min)		
	CER	FER	DC	CER	FER	DC
Time (min.)	13.71	70.98	240.0	11.56	36.57	120.0
Accumulated extract (%)	15.76	21.44	24.28	16.70	21.16	24.10
Total extract recovery (%)	55.68	86.08	100.0	66.57	86.09	100.0
M (g extract/min)	0.039	5.0 × 10^−3^	7.9 × 10^−4^	0.056	7.4 × 10^−3^	1.6 × 10^−3^
Y (mg extract/g biomass)	135.50	70.80	33.39	159.37	46.87	33.21
Y (mg astaxanthin/g biomass)	2.78	10.45	10.58	6.75	8.50	9.42
Total Recovery of astaxanthin (%)	11.91	56.72	100.0	28.95	65.43	100.0
Y* (g extract/g CO_2_)	1.1 × 10^−2^	1.4 × 10^−3^	2.2 × 10^−4^	7.7 × 10^−3^	1.0 × 10^−3^	2.2 × 10^−4^
R^2^	0.9904	1.0000	1.0000	0.9436	1.0000	1.0000

$t_{\mathrm{CER}},\;\mathrm{and}\;t_{\mathrm{FER}}:$ Times in the intercepts of the lines 1 y 2 and the lines 2 and 3, respectively; $m_{\mathrm{EXT}}\left(t\right):$ mass of the extract at time t.
